# *R248G cystic fibrosis transmembrane conductance regulator* mutation in three siblings presenting with recurrent acute pancreatitis and reproductive issues: a case series

**DOI:** 10.1186/s13256-016-1181-3

**Published:** 2017-02-15

**Authors:** Seiichi Villalona, Guillermo Glover-López, Juan Antonio Ortega-García, Rosa Moya-Quiles, Pedro Mondejar-López, Maria C. Martínez-Romero, Mariano Rigabert-Montiel, María D. Pastor-Vivero, Manuel Sánchez-Solís

**Affiliations:** 1 Pediatric Environmental Health Specialty Unit, University Hospital Virgen of Arrixaca, Murcia, Spain; 2 Center of Clinical Genetics, University Hospital Virgen of Arrixaca, Murcia, Spain; 3 Cystic Fibrosis Unit, Pediatric Neumology, University Hospital Virgen of Arrixaca, Murcia, Spain; 4 Urology & Andrology, University Hospital Virgen of Arrixaca, Murcia, Spain

**Keywords:** CFTR, Missense mutation, Genotype-phenotype, Congenital absence of vas deferens

## Abstract

**Background:**

Mutational combinations of the *cystic fibrosis transmembrane conductance regulator*, *CFTR*, gene have different phenotypic manifestations at the molecular level with varying clinical consequences for individuals possessing such mutations. Reporting *cystic fibrosis transmembrane conductance regulator* mutations is important in understanding the genotype-phenotype correlations and associated clinical presentations in patients with cystic fibrosis. Understanding the effects of mutations is critical in developing appropriate treatments for individuals affected with cystic fibrosis, non-classic cystic fibrosis, or cystic fibrosis transmembrane conductance regulator-related disorders. This is the first report of related individuals possessing the R248G missense *cystic fibrosis transmembrane conductance regulator* mutation and we present their associated clinical histories.

**Case presentation:**

All three patients are of Spanish descent. Deoxyribonucleic acid analysis revealed that all three siblings possessed a novel c.742A>G mutation, resulting in a p.Arg248Gly (R248G) amino acid change in exon 6 *in trans* with the known N1303K mutant allele. Case 1 patient is a 39-year-old infertile man presenting with congenital unilateral absence of the vas deferens and recurrent episodes of epigastric pain. Case 2 patient is a 32-year-old woman presenting with periods of infertility, two previous spontaneous abortions, recurrent epigastric pain, and recurrent pancreatitis. Case 3 patient is a 29-year-old woman presenting with recurrent pancreatitis and epigastric pain.

**Conclusions:**

We report the genotype-phenotype correlations and clinical manifestations of a novel R248G *cystic fibrosis transmembrane conductance regulator* mutation: congenital unilateral absence of the vas deferens in males, reduced female fertility, and recurrent acute pancreatitis. In addition, we discuss the possible functional consequences of the mutations at the molecular level.

**Electronic supplementary material:**

The online version of this article (doi:10.1186/s13256-016-1181-3) contains supplementary material, which is available to authorized users.

## Background

Cystic fibrosis (CF), Online Mendelian Inheritance in Man (OMIM) #219700, is a life-threatening autosomal recessive monogenetic disease caused by mutations to the *cystic fibrosis transmembrane conductance regulator* (*CFTR*) gene (OMIM #602421). CFTR is an adenosine triphosphate (ATP)-binding cassette (ABC) transporter protein that functions as a chloride (Cl^–^) ion channel and is expressed in the apical membrane of epithelial cells lining the lungs, pancreas, gastrointestinal tract, and reproductive system [1]. Transport of Cl^–^ ions is important in the movement of fluids required for the production of free flowing mucus in these epithelia [1]. Mutations to the *CFTR* gene phenotypically manifest themselves in variable clinical outcomes including: progressive decrease in lung function, recurrent colonization of opportunistic bacteria such as *Pseudomonas aeruginosa* and *Staphylococcus aureus*, pancreatic insufficiency (PI), and male infertility due to congenital bilateral absence of the vas deferens (CBAVD) or congenital unilateral absence of the vas deferens (CUAVD) [2, 3]. Accurate reporting of *CFTR* mutations is of clinical interest in improving genetic screening techniques, understanding CF genotype-phenotype correlations, and treatments of individuals affected with CF, non-classic CF, or CFTR-related disorders (CFTR-RD).

**Table 1:** Clinical expression of three siblings with the N1303K/R248G *cystic fibrosis transmembrane conductance regulator* genotype

	Patient 1	Patient 2	Patient 3
Current age	39-year-old man	32-year-old woman	29-year-old woman
Age of diagnosis	32 years	25 years	22 years
Chloride sweat test (mEq/L)	47	40	42
FEV1 %	82.2	86	84
FVC %	80.1	82	82.4
Respiratory disease	No significant history	No significant history	1 episode of bronchitis
			1 episode of pneumonia
Bacterial colonization	*Staphylococcus aureus*	*Staphylococcus aureus*	No significant history
Pancreatic and gastrointestinal manifestations	• Recurrent epigastric pain associated with pancreatitis	• 3 episodes of pancreatitis	• 4 episodes of pancreatitis
	• Chronic heartburn	• Recurrent epigastric pain	
Reproductive complications	• CUAVD	• 2 spontaneous abortions	No significant history
	• Testicular hydrocele	• Periods of infertility	

*CUAVD* congenital unilateral absence of the vas deferens, *FEV1* forced expiratory volume in 1 second, *FVC* forced vital capacity

We present here the case of three related Spanish patients possessing a novel CFTR R248G missense mutation *in trans* with the N1303K mutant allele. The hospital network ethics committees and the institutional review boards approved the study. Informed consent was obtained from the patients. Molecular studies of genomic deoxyribonucleic acid (DNA) were performed from extracted peripheral blood samples of the patients. In the index case, the use of the “Cystic Fibrosis Genotyping assay” (Abbott) kit identified a c.3909C>G mutation responsible for a p.Asn1303Lys change. After clinical evaluation was carried out, the sequencing of exons and adjoining regions of the *CFTR* gene were conducted by direct sequencing (3130X ABI PRISM sequencer; Applied Biosystems, Foster City, CA, USA). The sequencing analysis confirmed the previous mutation and identified a c.742A>G mutation, resulting in a p.Arg248Gly amino acid change in exon 6 of the *CFTR* gene. This new mutation was passed paternally and is present in all three siblings.

## Case presentation

Case 1 patient is a 39-year-old infertile man of Spanish descent who was referred to our CF Unit for non-classic CF evaluation after consulting with his primary care physician with regards to an inability to impregnate a woman in 2 years. A clinical evaluation confirmed the diagnosis of CUAVD on his left testicle and an in-depth past medical history was obtained. His history revealed that he had previously undergone surgical repair for right testicular hydrocele, recurrent episodes of acute pancreatitis with heartburn, and epigastric pain associated with increasing blood levels of amylase and lipase. Other potential causes of acute pancreatitis were ruled out (gallstones, endocrine problems and metabolic abnormalities, alcohol abuse, or others drugs). Pulmonary function tests suggested that case 1 patient does not have lung disease, although he has tested positive for *S. aureus* colonization. His sweat test averages of 47 mEq/l indicate borderline results for CF diagnosis. A review of his past clinical history was unremarkable for other CFTR-RDs. A non-classic CF clinical evaluation and evaluation of the past medical histories of his siblings were conducted as a means of examining particular genetic associations.

Case 2 patient is a 32-year-old Spanish woman whose past medical history was significant for periods of infertility with two previous spontaneous abortions. Her history also included recurrent episodes of epigastric pain and pancreatitis. The episodes of pancreatitis consisted of periodic acute periumbilical abdominal pain, vomiting, nausea, as well as a transient increase in enzymatic levels of amylase and pancreatic lipase. Similar to case 1, she had no prior medical or socio-behavioral histories that could account for the recurrent episodes of epigastric pain and pancreatitis. She exhibited normal lung function and has tested positive for *S. aureus* colonization. Her sweat test averages of 40 mEq/l indicate borderline results for CF diagnosis.

Case 3 patient is a 29-year-old Spanish woman with a past medical history of recurrent pancreatitis and epigastric pain. Her presentations of recurrent epigastric pain resembled that of the clinical history of case 1. Her episodes of recurrent pancreatitis were similar to the etiologies of case 2 with acute periumbilical abdominal pain, vomiting, nausea, as well as a transient increase in the enzymatic levels of amylase and pancreatic lipase. All other medical and socio-behavioral histories were also ruled out as potential causes of recurrent pancreatitis and epigastric pain. Her clinical history indicated previous periods of pneumonia and bronchitis. She exhibited normal lung function, has tested negative for *S. aureus* colonization, and has no prior history of reproductive problems. Her sweat test averages of 42 mEq/l indicate borderline results for CF diagnosis. Table 1 shows the summary of the clinical histories.

## Discussion

**Fig. 1:**
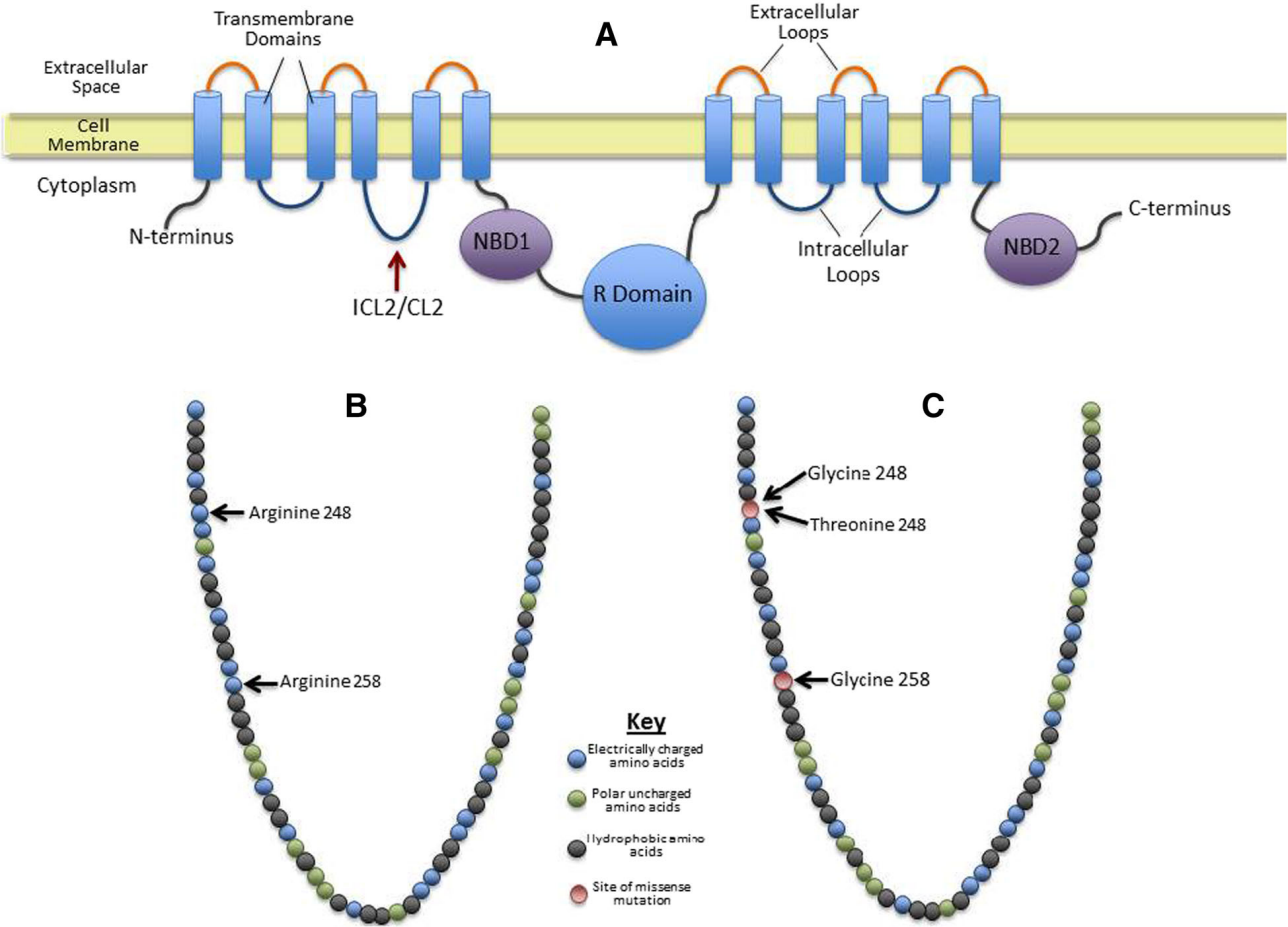
*Cystic fibrosis transmembrane conductance regulator* protein. **a** Two-dimensional model of the *cystic fibrosis transmembrane conductance regulator* protein with major functional components. **b** Wild-type intracellular loop 2/cellular loop 2 primary protein structure depicting amino acid properties. **c** Intracellular loop 2/cellular loop 2 regions clinically associated with congenital bilateral absence of the vas deferens or congenital unilateral absence of the vas deferens resulting from a reported missense mutation. *ICL2/CL2* intracellular loop 2/cellular loop 2, *NBD1* nucleotide-binding domain 1, *NBD2* nucleotide-binding domain 2

The N1303K allele is a product of a missense mutation in exon 21 of the *CFTR* gene [3], causing an asparagine to lysine amino acid change in the nucleotide-binding domain 2 (NBD2) of the CFTR protein (Cystic Fibrosis Mutation Database, http://www.genet.sickkids.on.ca/cftr). NBD2 is an ATPase domain of the CFTR protein that is involved in closing of the ion channel via the energy produced from ATP hydrolysis [4]. NBD2 mutations can result in destabilization of channel activity and significant reductions in channel permeability [4]. In addition, N1303K is a class II trafficking mutation that can cause folding or deficiency of protein maturation and consequently result in absence or reduced quantity of CFTR protein [1, 5]. This mutation has been heterogeneously associated with CBAVD and CUAVD in males [6, 7] and moderate to severe PI [8]. Figure 1a illustrates the two-dimensional model of the CFTR protein and major functional regions.

The clinical phenotype of CUAVD in case 1 patient is consistent with previous studies of the N1303K mutation. Although correlations between CFTR mutations and reproduction have been thoroughly investigated in male patients, studies also suggest that CFTR mutations can result in reduced fertility in females due to the production of thick cervical mucus, and increase the likelihood of spontaneous abortions [9]. We propose that the N1303K mutation is responsible for the periods of infertility in case 2 patient and could also be related to her two spontaneous abortions. The recurrent pancreatic problems observed in all three patients may not be associated with the N1303K mutation despite its classification as a moderate to severe PI mutation [8]. Recurrent acute pancreatitis is observed in significantly higher frequencies among patients with CF carrying one or two mild PI mutations than patients carrying only one moderate to severe PI mutation [8].

All three patients in this case study possess a novel R248G missense mutation in exon 6 of the *CFTR* gene, causing an arginine to glycine amino acid change. Arginine-248 is located in intracellular loop 2/cellular loop 2 (ICL2/CL2) of the wild-type CFTR protein (http://www.uniprot.org). Figure 1b illustrates the wild-type primary protein structure based on the properties of the amino acids. ICL2/CL2 is both the largest and most hydrophilic loop of the CFTR protein that stabilizes the full conductance state of the channel [10]. Increased mean closed times are associated with ICL2/CL2 mutations of the CFTR protein [10].

In this study we conducted an extensive database and literature review, where the R248G mutation is not reported as an identified CFTR mutation. A similar missense mutation, R248T, has been previously reported as a mild CFTR-RD mutation that is associated with CBAVD and no other clinical phenotype (see Fig. 1c) [11]. The R248G mutation may alter the normal ICL2/CL2 function more than the R248T mutation based on the clinical phenotypes of the three patients. An arginine to threonine amino acid substitution alters residue charge from positive to neutral but does not change polarity. An arginine to glycine substitution changes the residue charge from positive to neutral as well as amino acid polarity. In addition, an R258G missense mutation located in the ICL2/CL2 domain was reported in a French male with CBAVD [12]. Based on these previous observations and three cases presented in this study, we suggest that amino acid substitution of arginine residues in the ICL2/CL2 domain of the CFTR protein with amino acids of contrasting molecular properties in charge and polarity could be associated with CBAVD and/or CUAVD (see Fig. 1c). The missense mutation on the ICL2/CL2 domain of the protein could result in increased mean closed times of normal CFTR function and contribute to increased thick epithelial viscosity of the reproductive tract of female patients. This could explain the clinical histories of spontaneous abortions and periods of infertility in case 2 patient. We also postulate that this mutation may have had an impact on the normal development of the male reproductive organs in case 1 patient that resulted in CUAVD. We have incorporated Additional files 1 and 2 to show the chromatogram and the amino acid sequence confirming the presence of the c.742A>G.

## Conclusions

We suggest that the R248G mutation may be associated with the recurrent acute pancreatitis exhibited by case 2 and case 3 patients because it is unlikely that the N1303K moderate to severe PI mutation is the underlying molecular cause when taking into consideration that each patient has only one copy of this mutant allele [8]. The recurrent episodes of epigastric pain and heartburn observed in case 1 and case 2 patients could be symptomatically masking the clinical signs of recurrent acute pancreatitis [13, 14].

In this case series we report the potential genotype-phenotype correlations with the novel R248G CFTR mutation: CUAVD in males, reduced female fertility, and recurrent acute pancreatitis. Future structure-functional studies on the CFTR protein can provide further insight on the impact of the R248G mutation at the molecular level.

## Additional files

Additional file 1: Direct sequencing of exon 6 of *cystic fibrosis transmembrane conductance regulator* gene by the standard Sanger method confirming the presence of the c.742A>G change in all three patients. A.) Representative chromatogram for the wild-type forward strand 5′ → 3′ sequence. B.) Representative chromatogram of the mutated forward strand 5′ → 3′ sequence. (DOCX 106 kb)

Additional file 2: Human *cystic fibrosis transmembrane conductance regulator* nucleotide and amino acid sequence. Unique nucleotide sequence GATGATGAAGTACA is present in wild-type cystic fibrosis transmembrane conductance regulator protein. GATGATGAAGTAACG sequence observed in all three patient cases is due to a c.742A>G mutation, resulting in codon change from AGA to GGA and consequent arginine to glycine change. (RTF 600 kb)

## Abbreviations

ATP: Adenosine triphosphate
CBAVD: Congenital bilateral absence of the vas deferens
CF: Cystic fibrosis
CFTR: Cystic fibrosis transmembrane conductance regulator
CFTR-RD: CFTR-related disorders
Cl^–^ ion: Chloride ion
CUAVD: Congenital unilateral absence of the vas deferens
ICL2/CL2: Intracellular loop 2/cellular loop 2
NBD2: Nucleotide-binding domain 2
OMIM: Online Mendelian Inheritance in Man
PI: Pancreatic insufficiency
