# Supplementary material for: R248G cystic fibrosis transmembrane conductance regulator mutation in three siblings presenting with recurrent acute pancreatitis and reproductive issues: a case series
Source: J Med Case Rep. 2017 Feb 15;11:42. doi: 10.1186/s13256-016-1181-3 (PMC5310058; doi:10.1186/s13256-016-1181-3)

**Additional file 1: Figure S1**

Direct sequencing of exon 6 of CFTR gene by the standard Sanger method confirming the presence of the c.742 A>G change in all 3 patients. A.) Representative chromatogram for the wild-type forward strand 5’→ 3’ sequence. B.) Representative chromatogram of the mutated forward strand 5’ → 3’ sequence.


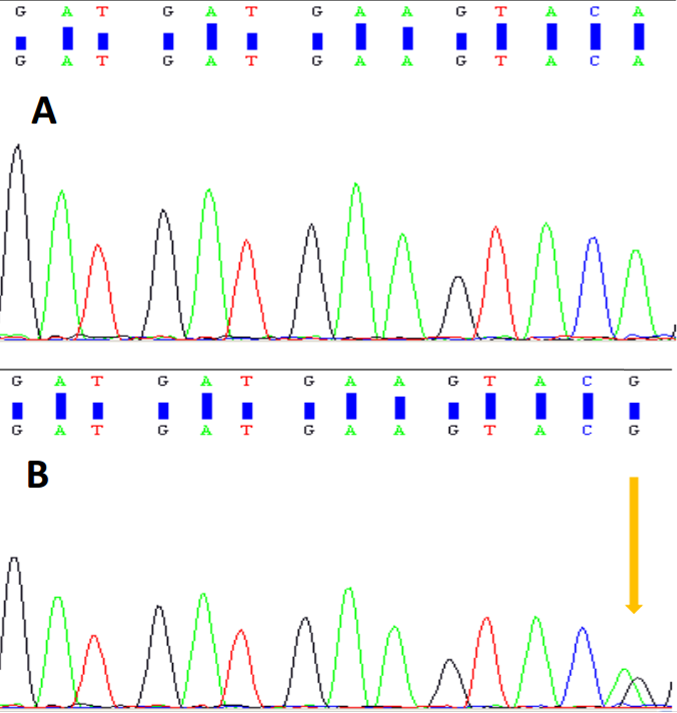

Supplement: Additional file 1: — Direct sequencing of exon 6 of cystic fibrosis transmembrane conductance regulator gene by the standard Sanger method confirming the presence of the c.742A>G change in all three patients. A.) Representative chromatogram for the wild-type forward strand 5′ → 3′ sequence. B.) Representative chromatogram of the mutated forward strand 5′ → 3′ sequence. (DOCX 106 kb) [file 13256_2016_1181_MOESM1_ESM.docx]
