# Supplementary material for: R248G cystic fibrosis transmembrane conductance regulator mutation in three siblings presenting with recurrent acute pancreatitis and reproductive issues: a case series
Source: J Med Case Rep. 2017 Feb 15;11:42. doi: 10.1186/s13256-016-1181-3 (PMC5310058; doi:10.1186/s13256-016-1181-3)
Supplement: Additional file 2: — Human cystic fibrosis transmembrane conductance regulator nucleotide and amino acid sequence. Unique nucleotide sequence GATGATGAAGTACA is present in wild-type cystic fibrosis transmembrane conductance regulator protein. GATGATGAAGTAACG sequence observed in all three patient cases is due to a c.742A>G mutation, resulting in codon change from AGA to GGA and consequent arginine to glycine change. (RTF 600 kb) [file 13256_2016_1181_MOESM2_ESM.rtf]

Additional file 2
Human CFTR nucleotide and amino acid sequence. Unique nucleotide sequence GATGATGAAGTACA is present in wild-type CFTR protein. GATGATGAAGTAACG sequence observed in all 3 patient cases is due to a c.742A>G mutation, resulting in codon change from AGA to GGA and consequent Arginine to Glycine change.

Retrieved from http://www.ensembl.org/Homo_sapiens/Transcript/Sequence_cDNA?db=core;g=ENSG00000001626;r=7:117479963-117668665;t=ENST00000003084

                     R Y  S       S                   M            
     1 AATTGGAAGCAAATGACATCACAGCAGGTCAGAGAAAAAGGGTTGAGCGGCAGGCACCCA
       ............................................................
       ............................................................
                                                            
                                             **   Y    R       Y   
    61 GAGTAGTAGGTCTTTGGCATTAGGAGCTTGAGCCCAGACGGCCCTAGCAGGGACCCCAGC
       ............................................................
       ............................................................
                                                            
          *S***S*B*RHDY*****KH**YY*  B    R   *R VY  Y  Y WW***    
   121 GCCCGAGAGACCATGCAGAGGTCGCCTCTGGAAAAGGCCAGCGTTGTCTCCAAACTTTTT
       ............ATGCAGAGGTCGCCTCTGGAAAAGGCCAGCGTTGTCTCCAAACTTTTT
       ............MQRSPLEKASVVSKLF
                                                            
       ***R    K  Y K   R     V   W  N*     S Y  HDMMY    *********
   181 TTCAGCTGGACCAGACCAATTTTGAGGAAAGGATACAGACAGCGCCTGGAATTGTCAGAC
    49 TTCAGCTGGACCAGACCAATTTTGAGGAAAGGATACAGACAGCGCCTGGAATTGTCAGAC
    17 FSWTRPILRKGYRQRLELSD
                                                            
       R*RHRSY*M  Y    Y VY  D     H      K YVYM R    *     *** R *
   241 ATATACCAAATCCCTTCTGTTGATTCTGCTGACAATCTATCTGAAAAATTGGAAAGAGAA
   109 ATATACCAAATCCCTTCTGTTGATTCTGCTGACAATCTATCTGAAAAATTGGAAAGAGAA
    37 IYQIPSVDSADNLSEKLERE
                                                            
       BRR******D V *       *   R  * *Y R WM*   W  YRM *  YR YD Y**
   301 TGGGATAGAGAGCTGGCTTCAAAGAAAAATCCTAAACTCATTAATGCCCTTCGGCGATGT
   169 TGGGATAGAGAGCTGGCTTCAAAGAAAAATCCTAAACTCATTAATGCCCTTCGGCGATGT
    57 WDRELASKKNPKLINALRRC
                                                            
       *  ***YR       W  **SYR  D R MH* *** R  Y R ** W  Y*Y     MW
   361 TTTTTCTGGAGATTTATGTTCTATGGAATCTTTTTATATTTAGGGGAAGTCACCAAAGCA
   229 TTTTTCTGGAGATTTATGTTCTATGGAATCTTTTTATATTTAGGGGAAGTCACCAAAGCA
    77 FFWRFMFYGIFLYLGEVTKA
                                                            
       K  YV  Y S   ***B *R * **W    **KYM*WRW* MSYRRR  W W  *W*RV 
   421 GTACAGCCTCTCTTACTGGGAAGAATCATAGCTTCCTATGACCCGGATAACAAGGAGGAA
   289 GTACAGCCTCTCTTACTGGGAAGAATCATAGCTTCCTATGACCCGGATAACAAGGAGGAA
    97 VQPLLLGRIIASYDPDNKEE
                                                            
       NS  **R *D*KW  YRW   S   Y RR  K **   *S  Y *R        W M   
   481 CGCTCTATCGCGATTTATCTAGGCATAGGCTTATGCCTTCTCTTTATTGTGAGGACACTG
   349 CGCTCTATCGCGATTTATCTAGGCATAGGCTTATGCCTTCTCTTTATTGTGAGGACACTG
   117 RSIAIYLGIGLCLLFIVRTL
                                                            
       *D******YY***M *    *   YW YR   S****R  *RHMSDK  R**Y*S**R**
   541 CTCCTACACCCAGCCATTTTTGGCCTTCATCACATTGGAATGCAGATGAGAATAGCTATG
   409 CTCCTACACCCAGCCATTTTTGGCCTTCATCACATTGGAATGCAGATGAGAATAGCTATG
   137 LLHPAIFGLHHIGMQMRIAM
                                                            
       ***H****   WDV W    *R   * RW  K   Y   HRWR  M   *****R  *S 
   601 TTTAGTTTGATTTATAAGAAGACTTTAAAGCTGTCAAGCCGTGTTCTAGATAAAATAAGT
   469 TTTAGTTTGATTTATAAGAAGACTTTAAAGCTGTCAAGCCGTGTTCTAGATAAAATAAGT
   157 FSLIYKKTLKLSSRVLDKIS
                                                            
        Y*RRRH  S    *R*YM Y*       M  MMY   M   K  RR*DR RKR     R
   661 ATTGGACAACTTGTTAGTCTCCTTTCCAACAACCTGAACAAATTTGATGAAGGACTTGCA
   529 ATTGGACAACTTGTTAGTCTCCTTTCCAACAACCTGAACAAATTTGATGAAGGACTTGCA
   177 IGQLVSLLSNNLNKFDEGLA
                                                            
       Y  VY YRKW M*    R  BR KYB  KKY   Y K RY*Y  YR   RRB***   RK
   721 TTGGCACATTTCGTGTGGATCGCTCCTTTGCAAGTGGCACTCCTCATGGGGCTAATCTGG
   589 TTGGCACATTTCGTGTGGATCGCTCCTTTGCAAGTGGCACTCCTCATGGGGCTAATCTGG
   197 LAHFVWIAPLQVALLMGLIW
                                                            
        R  W    YRR YR  Y   * SY W R  K   Y  YS R    W S  KYY YK Y 
   781 GAGTTGTTACAGGCGTCTGCCTTCTGTGGACTTGGTTTCCTGATAGTCCTTGCCCTTTTT
   649 GAGTTGTTACAGGCGTCTGCCTTCTGTGGACTTGGTTTCCTGATAGTCCTTGCCCTTTTT
   217 ELLQASAFCGLGFLIVLALF
                                                            
       Y S Y*R SS *RR**R*RYRRW******R****V          SY        W R  
   841 CAGGCTGGGCTAGGGAGAATGATGATGAAGTACAGAGATCAGAGAGCTGGGAAGATCAGT
   709 CAGGCTGGGCTAGGGAGAATGATGATGAAGTACAGAGATCAGAGAGCTGGGAAGATCAGT
   237 QAGLGRMMMKYRDQRAGKIS
                                                            
       *M R* *   K             MK        * *YY  R    Y  W      S  M
   901 GAAAGACTTGTGATTACCTCAGAAATGATTGAAAATATCCAATCTGTTAAGGCATACTGC
   769 GAAAGACTTGTGATTACCTCAGAAATGATTGAAAATATCCAATCTGTTAAGGCATACTGC
   257 ERLVITSEMIENIQSVKAYC
                                                            
       WR    R*WK  RY   W R*** WY  *********  Y   B K  M   ** ****Y
   961 TGGGAAGAAGCAATGGAAAAAATGATTGAAAACTTAAGACAAACAGAACTGAAACTGACT
   829 TGGGAAGAAGCAATGGAAAAAATGATTGAAAACTTAAGACAAACAGAACTGAAACTGACT
   277 WEEAMEKMIENLRQTELKLT
                                                            
       HR    * W SM R         BK Y RY R    RSN** * S*****RSD  YY***
  1021 CGGAAGGCAGCCTATGTGAGATACTTCAATAGCTCAGCCTTCTTCTTCTCAGGGTTCTTT
   889 CGGAAGGCAGCCTATGTGAGATACTTCAATAGCTCAGCCTTCTTCTTCTCAGGGTTCTTT
   297 RKAAYVRYFNSSAFFFSGFF
                                                            
        *   R   KWWY KR*     YS R   R *R     *KR  W    Y  YDR  *** 
  1081 GTGGTGTTTTTATCTGTGCTTCCCTATGCACTAATCAAAGGAATCATCCTCCGGAAAATA
   949 GTGGTGTTTTTATCTGTGCTTCCCTATGCACTAATCAAAGGAATCATCCTCCGGAAAATA
   317 VVFLSVLPYALIKGIILRKI
                                                            
       K  RYM * ******    *** Y  YYY YR *W  YR    B*YR Y V  YYY *SR
  1141 TTCACCACCATCTCATTCTGCATTGTTCTGCGCATGGCGGTCACTCGGCAATTTCCCTGG
  1009 TTCACCACCATCTCATTCTGCATTGTTCTGCGCATGGCGGTCACTCGGCAATTTCCCTGG
   337 FTTISFCIVLRMAVTRQFPW
                                                            
       *    RMRR*NR* * MN  YYY *Y   RR       Y  ***R   R *     M **
  1201 GCTGTACAAACATGGTATGACTCTCTTGGAGCAATAAACAAAATACAGGATTTCTTACAA
  1069 GCTGTACAAACATGGTATGACTCTCTTGGAGCAATAAACAAAATACAGGATTTCTTACAA
   357 AVQTWYDSLGAINKIQDFLQ
                                                            
       **  R D M R    M   Y  ****** S*  RYDW**R      BR*   K    W  
  1261 AAGCAAGAATATAAGACATTGGAATATAACTTAACGACTACAGAAGTAGTGATGGAGAAT
  1129 AAGCAAGAATATAAGACATTGGAATATAACTTAACGACTACAGAAGTAGTGATGGAGAAT
   377 KQEYKTLEYNLTTTEVVMEN
                                                            
         **   NY    DV     VS      RRRW ** W YS  R  ***** YM***  R 
  1321 GTAACAGCCTTCTGGGAGGAGGGATTTGGGGAATTATTTGAGAAAGCAAAACAAAACAAT
  1189 GTAACAGCCTTCTGGGAGGAGGGATTTGGGGAATTATTTGAGAAAGCAAAACAAAACAAT
   397 VTAFWEEGFGELFEKAKQNN
                                                            
         M RY K R      Y   WR       Y   M    Y  YR     **M*M**Y    
  1381 AACAATAGAAAAACTTCTAATGGTGATGACAGCCTCTTCTTCAGTAATTTCTCACTTCTT
  1249 AACAATAGAAAAACTTCTAATGGTGATGACAGCCTCTTCTTCAGTAATTTCTCACTTCTT
   417 NNRKTSNGDDSLFFSNFSLL
                                                            
        R R  Y*  Y  Y  **K **B**     **  K S      *  M******  HDKY 
  1441 GGTACTCCTGTCCTGAAAGATATTAATTTCAAGATAGAAAGAGGACAGTTGTTGGCGGTT
  1309 GGTACTCCTGTCCTGAAAGATATTAATTTCAAGATAGAAAGAGGACAGTTGTTGGCGGTT
   437 GTPVLKDINFKIERGQLLAV
                                                            
        ******YY*         * W K M  N YY  Y R DR*R******** S        
  1501 GCTGGATCCACTGGAGCAGGCAAGACTTCACTTCTAATGGTGATTATGGGAGAACTGGAG
  1369 GCTGGATCCACTGGAGCAGGCAAGACTTCACTTCTAATGGTGATTATGGGAGAACTGGAG
   457 AGSTGAGKTSLLMVIMGELE
                                                            
       Y   **K SDRY*****    Y* WS *   R   * M *KYYK  Y YM   **Y  RS
  1561 CCTTCAGAGGGTAAAATTAAGCACAGTGGAAGAATTTCATTCTGTTCTCAGTTTTCCTGG
  1429 CCTTCAGAGGGTAAAATTAAGCACAGTGGAAGAATTTCATTCTGTTCTCAGTTTTCCTGG
   477 PSEGKIKHSGRISFCSQFSW
                                                            
       *    SS   RMR  RH   *B   * VBV***BB*   R   Y    KR   MY YR  
  1621 ATTATGCCTGGCACCATTAAAGAAAATATCATCTTTGGTGTTTCCTATGATGAATATAGA
  1489 ATTATGCCTGGCACCATTAAAGAAAATATCATCTTTGGTGTTTCCTATGATGAATATAGA
   497 IMPGTIKENIIFGVSYDEYR
                                                            
        *      YN Y* S      Y MY     SRRR DSRYV S*  R  Y   M   KD  
  1681 TACAGAAGCGTCATCAAAGCATGCCAACTAGAAGAGGACATCTCCAAGTTTGCAGAGAAA
  1549 TACAGAAGCGTCATCAAAGCATGCCAACTAGAAGAGGACATCTCCAAGTTTGCAGAGAAA
   517 YRSVIKACQLEEDISKFAEK
                                                            
        *H    **R  M  KR  *RRKK  **K*R*W** MDKD *R* Y *BS*** R MR  
  1741 GACAATATAGTTCTTGGAGAAGGTGGAATCACACTGAGTGGAGGTCAACGAGCAAGAATT
  1609 GACAATATAGTTCTTGGAGAAGGTGGAATCACACTGAGTGGAGGTCAACGAGCAAGAATT
   537 DNIVLGEGGITLSGGQRARI
                                                            
       W*  Y*RH RVM** V  D***R*KR R* R   WKBRH   YY*R S Y YM  W KB 
  1801 TCTTTAGCAAGAGCAGTATACAAAGATGCTGATTTGTATTTATTAGACTCTCCTTTTGGA
  1669 TCTTTAGCAAGAGCAGTATACAAAGATGCTGATTTGTATTTATTAGACTCTCCTTTTGGA
   557 SLARAVYKDADLYLLDSPFG
                                                            
        WY  R** ***** WBMR     K  *  D  RD  N        R     * D***M 
  1861 TACCTAGATGTTTTAACAGAAAAAGAAATATTTGAAAGCTGTGTCTGTAAACTGATGGCT
  1729 TACCTAGATGTTTTAACAGAAAAAGAAATATTTGAAAGCTGTGTCTGTAAACTGATGGCT
   577 YLDVLTEKEIFESCVCKLMA
                                                            
        MBW***SWR RWY    DYY B YY ******RR**W*WY***S***R**KR****R**
  1921 AACAAAACTAGGATTTTGGTCACTTCTAAAATGGAACATTTAAAGAAAGCTGACAAAATA
  1789 AACAAAACTAGGATTTTGGTCACTTCTAAAATGGAACATTTAAAGAAAGCTGACAAAATA
   597 NKTRILVTSKMEHLKKADKI
                                                            
       **W*Y**Y**NKR***R*******W****K***V*R******Y*****MYYY      Y 
  1981 TTAATTTTGCATGAAGGTAGCAGCTATTTTTATGGGACATTTTCAGAACTCCAAAATCTA
  1849 TTAATTTTGCATGAAGGTAGCAGCTATTTTTATGGGACATTTTCAGAACTCCAAAATCTA
   617 LILHEGSSYFYGTFSELQNL
                                                            
       Y **  K   *YR *Y******YS H   W   RW   Y  HR  V     R     K  
  2041 CAGCCAGACTTTAGCTCAAAACTCATGGGATGTGATTCTTTCGACCAATTTAGTGCAGAA
  1909 CAGCCAGACTTTAGCTCAAAACTCATGGGATGTGATTCTTTCGACCAATTTAGTGCAGAA
   637 QPDFSSKLMGCDSFDQFSAE
                                                            
           R*****S**********K  *   K   YYR     *****R* K   D    B Y
  2101 AGAAGAAATTCAATCCTAACTGAGACCTTACACCGTTTCTCATTAGAAGGAGATGCTCCT
  1969 AGAAGAAATTCAATCCTAACTGAGACCTTACACCGTTTCTCATTAGAAGGAGATGCTCCT
   657 RRNSILTETLHRFSLEGDAP
                                                            
           Y  R    SW *** R ****   M  ***  * RR    WK  Y*** ***  R*
  2161 GTCTCCTGGACAGAAACAAAAAAACAATCTTTTAAACAGACTGGAGAGTTTGGGGAAAAA
  2029 GTCTCCTGGACAGAAACAAAAAAACAATCTTTTAAACAGACTGGAGAGTTTGGGGAAAAA
   677 VSWTETKKQSFKQTGEFGEK
                                                            
       ** R*K  W        S WY Y R   W     Y Y* W      *   WK  Y MW K
  2221 AGGAAGAATTCTATTCTCAATCCAATCAACTCTATACGAAAATTTTCCATTGTGCAAAAG
  2089 AGGAAGAATTCTATTCTCAATCCAATCAACTCTATACGAAAATTTTCCATTGTGCAAAAG
   697 RKNSILNPINSIRKFSIVQK
                                                            
        Y  S  W*YR        K   Y*****      K   K   Y  K       *R    
  2281 ACTCCCTTACAAATGAATGGCATCGAAGAGGATTCTGATGAGCCTTTAGAGAGAAGGCTG
  2149 ACTCCCTTACAAATGAATGGCATCGAAGAGGATTCTGATGAGCCTTTAGAGAGAAGGCTG
   717 TPLQMNGIEEDSDEPLERRL
                                                            
       WY    *   Y             K      YR***Y***Y*YVY*K  RBR RW     
  2341 TCCTTAGTACCAGATTCTGAGCAGGGAGAGGCGATACTGCCTCGCATCAGCGTGATCAGC
  2209 TCCTTAGTACCAGATTCTGAGCAGGGAGAGGCGATACTGCCTCGCATCAGCGTGATCAGC
   737 SLVPDSEQGEAILPRISVIS
                                                            
              ** BR Y Y K   Y*  R  D               Y        WYR* S 
  2401 ACTGGCCCCACGCTTCAGGCACGAAGGAGGCAGTCTGTCCTGAACCTGATGACACACTCA
  2269 ACTGGCCCCACGCTTCAGGCACGAAGGAGGCAGTCTGTCCTGAACCTGATGACACACTCA
   757 TGPTLQARRRQSVLNLMTHS
                                                            
                   Y    MRK    YV   S ** * K       RBR        *    
  2461 GTTAACCAAGGTCAGAACATTCACCGAAAGACAACAGCATCCACACGAAAAGTGTCACTG
  2329 GTTAACCAAGGTCAGAACATTCACCGAAAGACAACAGCATCCACACGAAAAGTGTCACTG
   777 VNQGQNIHRKTTASTRKVSL
                                                            
        ****    KS   Y       W     R * R***   R    K** *  Y  K  *  
  2521 GCCCCTCAGGCAAACTTGACTGAACTGGATATATATTCAAGAAGGTTATCTCAAGAAACT
  2389 GCCCCTCAGGCAAACTTGACTGAACTGGATATATATTCAAGAAGGTTATCTCAAGAAACT
   797 APQANLTELDIYSRRLSQET
                                                            
        K  **     W  *D  K W  *  YR MK  R*    W RKR KRMY   *** YK *
  2581 GGCTTGGAAATAAGTGAAGAAATTAACGAAGAAGACTTAAAGGAGTGCTTTTTTGATGAT
  2449 GGCTTGGAAATAAGTGAAGAAATTAACGAAGAAGACTTAAAGGAGTGCTTTTTTGATGAT
   817 GLEISEEINEEDLKECFFDD
                                                            
        Y   R    * YSW  R          RR R   WYRM   YD*** D Y Y*RY** Y
  2641 ATGGAGAGCATACCAGCAGTGACTACATGGAACACATACCTTCGATATATTACTGTCCAC
  2509 ATGGAGAGCATACCAGCAGTGACTACATGGAACACATACCTTCGATATATTACTGTCCAC
   837 MESIPAVTTWNTYLRYITVH
                                                            
         V     MMK   *K*   *******YR***** RR         M   *S        
  2701 AAGAGCTTAATTTTTGTGCTAATTTGGTGCTTAGTAATTTTTCTGGCAGAGGTGGCTGCT
  2569 AAGAGCTTAATTTTTGTGCTAATTTGGTGCTTAGTAATTTTTCTGGCAGAGGTGGCTGCT
   857 KSLIFVLIWCLVIFLAEVAA
                                                            
       K    *******Y   R Y   W   *   MM  M Y  YR RRY   R R    V  Y*
  2761 TCTTTGGTTGTGCTGTGGCTCCTTGGAAACACTCCTCTTCAAGACAAAGGGAATAGTACT
  2629 TCTTTGGTTGTGCTGTGGCTCCTTGGAAACACTCCTCTTCAAGACAAAGGGAATAGTACT
   877 SLVVLWLLGNTPLQDKGNST
                                                            
       Y*         W  Y  SYR          RKY M  K     R  H***W  YS  W  
  2821 CATAGTAGAAATAACAGCTATGCAGTGATTATCACCAGCACCAGTTCGTATTATGTGTTT
  2689 CATAGTAGAAATAACAGCTATGCAGTGATTATCACCAGCACCAGTTCGTATTATGTGTTT
   897 HSRNNSYAVIITSTSSYYVF
                                                            
       KR   K R D *RR***R MYRR  Y***K Y              * R WR    RM R
  2881 TACATTTACGTGGGAGTAGCCGACACTTTGCTTGCTATGGGATTCTTCAGAGGTCTACCA
  2749 TACATTTACGTGGGAGTAGCCGACACTTTGCTTGCTATGGGATTCTTCAGAGGTCTACCA
   917 YIYVGVADTLLAMGFFRGLP
                                                            
       ***BK SR**YK *  *     YD YRW   K  Y YDY   R   YV  RYM****S**
  2941 CTGGTGCATACTCTAATCACAGTGTCGAAAATTTTACACCACAAAATGTTACATTCTGTT
  2809 CTGGTGCATACTCTAATCACAGTGTCGAAAATTTTACACCACAAAATGTTACATTCTGTT
   937 LVHTLITVSKILHHKMLHSV
                                                            
       Y******Y*Y***YK****H**    M**R Y      VV*  RR  WYH     *  YM
  3001 CTTCAAGCACCTATGTCAACCCTCAACACGTTGAAAGCAGGTGGGATTCTTAATAGATTC
  2869 CTTCAAGCACCTATGTCAACCCTCAACACGTTGAAAGCAGGTGGGATTCTTAATAGATTC
   957 LQAPMSTLNTLKAGGILNRF
                                                            
       BY W  RH  W*     K*  R  N M Y        K***YR R YWKR  KMM  Y *
  3061 TCCAAAGATATAGCAATTTTGGATGACCTTCTGCCTCTTACCATATTTGACTTCATCCAG
  2929 TCCAAAGATATAGCAATTTTGGATGACCTTCTGCCTCTTACCATATTTGACTTCATCCAG
   977 SKDIAILDDLLPLTIFDFIQ
                                                            
         S*********V**W**KR**S**K*RM** **WYRY      R  R ***R*  Y Y 
  3121 TTGTTATTAATTGTGATTGGAGCTATAGCAGTTGTCGCAGTTTTACAACCCTACATCTTT
  2989 TTGTTATTAATTGTGATTGGAGCTATAGCAGTTGTCGCAGTTTTACAACCCTACATCTTT
   997 LLLIVIGAIAVVAVLQPYIF
                                                            
          R  R  RW H *R***K*****Y    WYWRKK  **  RYRWR K Y   Y  *MY
  3181 GTTGCAACAGTGCCAGTGATAGTGGCTTTTATTATGTTGAGAGCATATTTCCTCCAAACC
  3049 GTTGCAACAGTGCCAGTGATAGTGGCTTTTATTATGTTGAGAGCATATTTCCTCCAAACC
  1017 VATVPVIVAFIMLRAYFLQT
                                                            
              R    M  Y   *  R  *    *D *     S  D  K   YKS*W*     
  3241 TCACAGCAACTCAAACAACTGGAATCTGAAGGCAGGAGTCCAATTTTCACTCATCTTGTT
  3109 TCACAGCAACTCAAACAACTGGAATCTGAAGGCAGGAGTCCAATTTTCACTCATCTTGTT
  1037 SQQLKQLESEGRSPIFTHLV
                                                            
       R     YKR MRS     ***   YB HR VNY  YR  YV YM* ** *   W   *M 
  3301 ACAAGCTTAAAAGGACTATGGACACTTCGTGCCTTCGGACGGCAGCCTTACTTTGAAACT
  3169 ACAAGCTTAAAAGGACTATGGACACTTCGTGCCTTCGGACGGCAGCCTTACTTTGAAACT
  1057 TSLKGLWTLRAFGRQPYFET
                                                            
       *Y*    M M**S          R R WY KY ****RS     SYRM Y   R  W * 
  3361 CTGTTCCACAAAGCTCTGAATTTACATACTGCCAACTGGTTCTTGTACCTGTCAACACTG
  3229 CTGTTCCACAAAGCTCTGAATTTACATACTGCCAACTGGTTCTTGTACCTGTCAACACTG
  1077 LFHKALNLHTANWFLYLSTL
                                                            
       YR*YR*  M M  DRWRR   K   K* Y    S  R Y   Y S    YYS      Y 
  3421 CGCTGGTTCCAAATGAGAATAGAAATGATTTTTGTCATCTTCTTCATTGCTGTTACCTTC
  3289 CGCTGGTTCCAAATGAGAATAGAAATGATTTTTGTCATCTTCTTCATTGCTGTTACCTTC
  1097 RWFQMRIEMIFVIFFIAVTF
                                                            
        Y  B          *  S ***** R    R W   *  S R Y      **    R  
  3481 ATTTCCATTTTAACAACAGGAGAAGGAGAAGGAAGAGTTGGTATTATCCTGACTTTAGCC
  3349 ATTTCCATTTTAACAACAGGAGAAGGAGAAGGAAGAGTTGGTATTATCCTGACTTTAGCC
  1117 ISILTTGEGEGRVGIILTLA
                                                            
       RBS   R  RW*  *** Y RY  * R M R R RM* M      SM RW KR     KD
  3541 ATGAATATCATGAGTACATTGCAGTGGGCTGTAAACTCCAGCATAGATGTGGATAGCTTG
  3409 ATGAATATCATGAGTACATTGCAGTGGGCTGTAAACTCCAGCATAGATGTGGATAGCTTG
  1137 MNIMSTLQWAVNSSIDVDSL
                                                            
       W RYR YY    M *YN* K ****R YK R   R       R  R  R   M     **
  3601 ATGCGATCTGTGAGCCGAGTCTTTAAGTTCATTGACATGCCAACAGAAGGTAAACCTACC
  3469 ATGCGATCTGTGAGCCGAGTCTTTAAGTTCATTGACATGCCAACAGAAGGTAAACCTACC
  1157 MRSVSRVFKFIDMPTEGKPT
                                                            
       W*R Y***Y* * M Y S       R Y RV  Y R   S*  Y           M  SR
  3661 AAGTCAACCAAACCATACAAGAATGGCCAACTCTCGAAAGTTATGATTATTGAGAATTCA
  3529 AAGTCAACCAAACCATACAAGAATGGCCAACTCTCGAAAGTTATGATTATTGAGAATTCA
  1177 KSTKPYKNGQLSKVMIIENS
                                                            
        D * K R M RK W * R Y RR    V**   *   RVW*   R            M 
  3721 CACGTGAAGAAAGATGACATCTGGCCCTCAGGGGGCCAAATGACTGTCAAAGATCTCACA
  3589 CACGTGAAGAAAGATGACATCTGGCCCTCAGGGGGCCAAATGACTGTCAAAGATCTCACA
  1197 HVKKDDIWPSGGQMTVKDLT
                                                            
        Y    Y ***            W Y  *  Y S*   M ***        N    K   
  3781 GCAAAATACACAGAAGGTGGAAATGCCATATTAGAGAACATTTCCTTCTCAATAAGTCCT
  3649 GCAAAATACACAGAAGGTGGAAATGCCATATTAGAGAACATTTCCTTCTCAATAAGTCCT
  1217 AKYTEGGNAILENISFSISP
                                                            
       *R HR W R K   H  Y   RD   R Y S   Y*RR*    RYM  YYV KWYSR***
  3841 GGCCAGAGGGTGGGCCTCTTGGGAAGAACTGGATCAGGGAAGAGTACTTTGTTATCAGCT
  3709 GGCCAGAGGGTGGGCCTCTTGGGAAGAACTGGATCAGGGAAGAGTACTTTGTTATCAGCT
  1237 GQRVGLLGRTGSGKSTLLSA
                                                            
       Y K****   *R**K   *YY*   R        R  *B*R*    K**    RR     
  3901 TTTTTGAGACTACTGAACACTGAAGGAGAAATCCAGATCGATGGTGTGTCTTGGGATTCA
  3769 TTTTTGAGACTACTGAACACTGAAGGAGAAATCCAGATCGATGGTGTGTCTTGGGATTCA
  1257 FLRLLNTEGEIQIDGVSWDS
                                                            
       *Y          BR B V N   R Y* Y *    R W HYRYRS  *R********* *
  3961 ATAACTTTGCAACAGTGGAGGAAAGCCTTTGGAGTGATACCACAGAAAGTATTTATTTTT
  3829 ATAACTTTGCAACAGTGGAGGAAAGCCTTTGGAGTGATACCACAGAAAGTATTTATTTTT
  1277 ITLQQWRKAFGVIPQKVFIF
                                                            
       *** SRRYRY   ***  ********WY**WRWK  B SKR    RR H  *******R 
  4021 TCTGGAACATTTAGAAAAAACTTGGATCCCTATGAACAGTGGAGTGATCAAGAAATATGG
  3889 TCTGGAACATTTAGAAAAAACTTGGATCCCTATGAACAGTGGAGTGATCAAGAAATATGG
  1297 SGTFRKNLDPYEQWSDQEIW
                                                            
           YY M**  R  D      Y    *     *YRS  S    WY  K     YY    
  4081 AAAGTTGCAGATGAGGTTGGGCTCAGATCTGTGATAGAACAGTTTCCTGGGAAGCTTGAC
  3949 AAAGTTGCAGATGAGGTTGGGCTCAGATCTGTGATAGAACAGTTTCCTGGGAAGCTTGAC
  1317 KVADEVGLRSVIEQFPGKLD
                                                            
       K     YY     R   K*** S    *******  RR    R RB B  S YRKK   R
  4141 TTTGTCCTTGTGGATGGGGGCTGTGTCCTAAGCCATGGCCACAAGCAGTTGATGTGCTTG
  4009 TTTGTCCTTGTGGATGGGGGCTGTGTCCTAAGCCATGGCCACAAGCAGTTGATGTGCTTG
  1337 FVLVDGGCVLSHGHKQLMCL
                                                            
         **S*  ****S   ***   YR  RWYY      Y YSR K  MY  K  S MV    
  4201 GCTAGATCTGTTCTCAGTAAGGCGAAGATCTTGCTGCTTGATGAACCCAGTGCTCATTTG
  4069 GCTAGATCTGTTCTCAGTAAGGCGAAGATCTTGCTGCTTGATGAACCCAGTGCTCATTTG
  1357 ARSVLSKAKILLLDEPSAHL
                                                            
       V   Y S   **YMMH *********        W    Y *   Y       Y   M  
  4261 GATCCAGTAACATACCAAATAATTAGAAGAACTCTAAAACAAGCATTTGCTGATTGCACA
  4129 GATCCAGTAACATACCAAATAATTAGAAGAACTCTAAAACAAGCATTTGCTGATTGCACA
  1377 DPVTYQIIRRTLKQAFADCT
                                                            
        W  K***B  *DV   Y R    R      *   RR*    YM Y RY   Y* W    
  4321 GTAATTCTCTGTGAACACAGGATAGAAGCAATGCTGGAATGCCAACAATTTTTGGTCATA
  4189 GTAATTCTCTGTGAACACAGGATAGAAGCAATGCTGGAATGCCAACAATTTTTGGTCATA
  1397 VILCEHRIEAMLECQQFLVI
                                                            
         *K K      K  YRR     YK  YH  Y    *    *Y******* **    Y  
  4381 GAAGAGAACAAAGTGCGGCAGTACGATTCCATCCAGAAACTGCTGAACGAGAGGAGCCTC
  4249 GAAGAGAACAAAGTGCGGCAGTACGATTCCATCCAGAAACTGCTGAACGAGAGGAGCCTC
  1417 EENKVRQYDSIQKLLNERSL
                                                            
       Y  Y         Y M M    YMR   R      R  MW  Y     BR    KS  K 
  4441 TTCCGGCAAGCCATCAGCCCCTCCGACAGGGTGAAGCTCTTTCCCCACCGGAACTCAAGC
  4309 TTCCGGCAAGCCATCAGCCCCTCCGACAGGGTGAAGCTCTTTCCCCACCGGAACTCAAGC
  1437 FRQAISPSDRVKLFPHRNSS
                                                            
         K       Y   K     R Y    K                    K     D RYR 
  4501 AAGTGCAAGTCTAAGCCCCAGATTGCTGCTCTGAAAGAGGAGACAGAAGAAGAGGTGCAA
  4369 AAGTGCAAGTCTAAGCCCCAGATTGCTGCTCTGAAAGAGGAGACAGAAGAAGAGGTGCAA
  1457 KCKSKPQIAALKEETEEEVQ
                                                            
         Y SR    Y  *  *      W R R   YR  R       YY  Y********Y***
  4561 GATACAAGGCTTTAGAGAGCAGCATAAATGTTGACATGGGACATTTGCTCATGGAATTGG
  4429 GATACAAGGCTTTAG.............................................
  1477 DTRL*.............................................
                                                            
       **Y**R************         Y      KY            Y           
  4621 AGCTCGTGGGACAGTCACCTCATGGAATTGGAGCTCGTGGAACAGTTACCTCTGCCTCAG
       ............................................................
       ............................................................
                                                            
                         **      WW          Y     R    R          
  4681 AAAACAAGGATGAATTAAGTTTTTTTTTAAAAAAGAAACATTTGGTAAGGGGAATTGAGG
       ............................................................
       ............................................................
                                                            
          Y K                                                      
  4741 ACACTGATATGGGTCTTGATAAATGGCTTCCTGGCAATAGTCAAATTGTGTGAAAGGTAC
       ............................................................
       ............................................................
                                                            
                                                               S   
  4801 TTCAAATCCTTGAAGATTTACCACTTGTGTTTTGCAAGCCAGATTTTCCTGAAAACCCTT
       ............................................................
       ............................................................
                                                            
                   W    S                     R              Y     
  4861 GCCATGTGCTAGTAATTGGAAAGGCAGCTCTAAATGTCAATCAGCCTAGTTGATCAGCTT
       ............................................................
       ............................................................
                                                            
                Y       R      W                                   
  4921 ATTGTCTAGTGAAACTCGTTAATTTGTAGTGTTGGAGAAGAACTGAAATCATACTTCTTA
       ............................................................
       ............................................................
                                                            
                                  W     Y  Y        W              
  4981 GGGTTATGATTAAGTAATGATAACTGGAAACTTCAGCGGTTTATATAAGCTTGTATTCCT
       ............................................................
       ............................................................
                                                            
         Y     S       Y                                           
  5041 TTTTCTCTCCTCTCCCCATGATGTTTAGAAACACAACTATATTGTTTGCTAAGCATTCCA
       ............................................................
       ............................................................
                                                            
                           R                                       
  5101 ACTATCTCATTTCCAAGCAAGTATTAGAATACCACAGGAACCACAAGACTGCACATCAAA
       ............................................................
       ............................................................
                                                            
        Y          Y   R               S         Y                 
  5161 ATATGCCCCATTCAACATCTAGTGAGCAGTCAGGAAAGAGAACTTCCAGATCCTGGAAAT
       ............................................................
       ............................................................
                                                            
                                           R R                     
  5221 CAGGGTTAGTATTGTCCAGGTCTACCAAAAATCTCAATATTTCAGATAATCACAATACAT
       ............................................................
       ............................................................
                                                            
                                          **                       
  5281 CCCTTACCTGGGAAAGGGCTGTTATAATCTTTCACAGGGGACAGGATGGTTCCCTTGATG
       ............................................................
       ............................................................
                                                            
                               R                                   
  5341 AAGAAGTTGATATGCCTTTTCCCAACTCCAGAAAGTGACAAGCTCACAGACCTTTGAACT
       ............................................................
       ............................................................
                                                            
                                                              Y    
  5401 AGAGTTTAGCTGGAAAAGTATGTTAGTGCAAATTGTCACAGGACAGCCCTTCTTTCCACA
       ............................................................
       ............................................................
                                                            
                 R                             R                M  
  5461 GAAGCTCCAGGTAGAGGGTGTGTAAGTAGATAGGCCATGGGCACTGTGGGTAGACACACA
       ............................................................
       ............................................................
                                                            
               M                                                   
  5521 TGAAGTCCAAGCATTTAGATGTATAGGTTGATGGTGGTATGTTTTCAGGCTAGATGTATG
       ............................................................
       ............................................................
                                                            
                            **   **     R   M  M                   
  5581 TACTTCATGCTGTCTACACTAAGAGAGAATGAGAGACACACTGAAGAAGCACCAATCATG
       ............................................................
       ............................................................
                                                            
                            Y  Y                                   
  5641 AATTAGTTTTATATGCTTCTGTTTTATAATTTTGTGAAGCAAAATTTTTTCTCTAGGAAA
       ............................................................
       ............................................................
                                                            
                                      W                            
  5701 TATTTATTTTAATAATGTTTCAAACATATATAACAATGCTGTATTTTAAAAGAATGATTA
       ............................................................
       ............................................................
                                                            
       Y  R                                                        
  5761 TGAATTACATTTGTATAAAATAATTTTTATATTTGAAATATTGACTTTTTATGGCACTAG
       ............................................................
       ............................................................
                                                            
          Y Y                                          R           
  5821 TATTTCTATGAAATATTATGTTAAAACTGGGACAGGGGAGAACCTAGGGTGATATTAACC
       ............................................................
       ............................................................
                                                            
                       S                    Y                      
  5881 AGGGGCCATGAATCACCTTTTGGTCTGGAGGGAAGCCTTGGGGCTGATGCAGTTGTTGCC
       ............................................................
       ............................................................
                                                            
                                 S     Y                           
  5941 CACAGCTGTATGATTCCCAGCCAGCACAGCCTCTTAGATGCAGTTCTGAAGAAGATGGTA
       ............................................................
       ............................................................
                                                            
                                  R                                
  6001 CCACCAGTCTGACTGTTTCCATCAAGGGTACACTGCCTTCTCAACTCCAAACTGACTCTT
       ............................................................
       ............................................................
                                                            
             R                *                                    
  6061 AAGAAGACTGCATTATATTTATTACTGTAAGAAAATATCACTTGTCAATAAAATCCATAC
       ............................................................
       ............................................................
                                                            
                 M 
  6121 ATTTGTGTGAAA
       ............
       ............
                                                            
